# Long-term osseointegration of laser-ablated hearing implants in sheep cranial bone

**DOI:** 10.3389/fsurg.2022.885964

**Published:** 2022-08-31

**Authors:** Martin Lars Johansson, Furqan A. Shah, Måns Eeg-Olofsson, Peter Monksfield, Peter Thomsen, Anders Palmquist

**Affiliations:** ^1^ Department of Biomaterials, Institute of Clinical Sciences, Sahlgrenska Academy, University of Gothenburg, Gothenburg, Sweden; ^2^ Research and Technology, Oticon Medical AB, Askim, Sweden; ^3^ Department of Otorhinolaryngology Head and Neck Surgery, Institute of Clinical Sciences, Sahlgrenska Academy, University of Gothenburg, Gothenburg, Sweden; ^4^ Department of Otorhinolaryngology, Head and Neck Surgery, Region Västra Götaland, Sahlgrenska University Hospital, Gothenburg, Sweden; ^5^ ENT Department, University Hospitals Birmingham NHS Foundation Trust, Queen Elizabeth Hospital Birmingham, Birmingham, United Kingdom

**Keywords:** osseointegration, bone anchored hearing, titanium implant, *in vivo*, laser, surface properties, hearing implant, BAHS

## Abstract

Osseointegration, the ability for an implant to be anchored in bone tissue with direct bone-implant contact and allowing for continuous adaptive remodelling, is clinically used in different reconstructive fields, such as dentistry, orthopedics and otology. The latter uses a bone conducting sound processor connected to a skin-penetrating abutment that is mounted on a titanium implant placed in the temporal bone, thereby acting as a path for transmission of the vibrations generated by the sound processor. The success of the treatment relies on bone healing and osseointegration, which could be improved by surface modifications. The aim of this study was to evaluate the long-term osseointegration in a sheep skull model and compare a laser-ablated implant surface with a machined implant. Commercially available 4 mm titanium implants, either with a machined (Wide Ponto) or a laser-ablated surface (Ponto BHX, Oticon Medical, Sweden), were used in the current study. The surfaces were evaluated by scanning electron microscopy. The implantation was performed with a full soft tissue flap and the osteotomy was prepared using the MIPS drill kit (Oticon Medical, Sweden) prior to installation of the implants in the frontal bone of eight female sheep. After five months, biopsies including the implant and surrounding bone tissue obtained, processed and analysed using histology, histomorphometry, scanning electron microscopy and Raman spectroscopy. The animals healed well, without signs of adverse events. Histomorphometry showed a large amount of bone tissue around both implant types, with 75% of the threaded area occupied by bone for both implant types. A large amount of bone-implant contact was observed for both implant types, with 67%–71% of the surface covered by bone. Both implant types were surrounded by mature remodelled lamellar bone with high mineral content, corroborating the histological observations. The current results show that the laser-ablated surface induces healing similar to the well-known clinically used machined surface in ovine cranial bone. In conclusion, the present long-term experimental results indicate that a laser-ablated implant performs equally well as a clinically used implant with a machined surface. This, together with previously reported, improved early biomechanical anchorage, suggests future, safe and efficient clinical potential.

## Introduction

Bone anchored hearing systems (BAHSs) are percutaneous bone conduction devices that have been used since the late 1970s. The sound is propagated to the inner ear by vibrations in the temporal bone and restores hearing over a wide frequency band for patients with mixed and conductive hearing loss and can also be important for improved speech perception in single sided deafness (1, 2). The concept relies on stable bone anchorage and osseointegration of the implant enabling the induction of vibrations and should last over long time periods, preferably surviving the life span of the patient.

It is well established that the surface morphology and chemistry of a titanium implant play an important role in the cellular response and osseointegration in bone. The first generation of titanium implants were typically machined (turned) with a relatively smooth, textured surface. Thereafter, a second generation of implant modifications emerged, for example, with blasted and acid-etched surfaces, in an attempt to accelerate and improve implant osseointegration (3, 4).

Intentional surface modification of a biomedical implant material is performed to promote biological reactions at the interface. In bone, these surface modifications are designed to influence the biological events that lead to bone formation, the close adaptation of mineralized bone to the material surface and an implant-bone shear strength that allows the implant to be loaded. Important key features of implant surface modifications are, first, that important bulk properties are retained and, second, that the positive biological reactions that are elicited persist, leading to maintained long-term integration and function. Most surface modifications of clinically available oral implants employ techniques that increase the roughness of the surface compared with the machined Ti surface, resulting in surface irregularities with different forms, shapes and sizes (4). Most of these roughened surfaces are produced either by blasting, abrading and coating methods using different material particles and/or by chemical methods. In a review of oral implants, moderately rough implants were considered to have the potential benefit of a “stronger bone response and tendency to better clinical results than turned implants” (5).

A third generation of surface modifications is now being introduced in clinical practice for various applications. One such example is a laser-ablated surface modification. By using laser technology, an oscillating laser beam locally creates a distinct surface structure with a combined macro, micro, and nanotopography (6). We have previously shown the improved bone anchorage and healing kinetics of a laser-modified implant surface in a rabbit model with 4 weeks of healing (7); however, a different implant design was used compared to the clinically used implant system.

The aim of the current study was to extend previous preclinical knowledge to a more clinically relevant model (sheep cranial bone) using a commercial implant design and to compare the long-term sheep skull bone tissue response in laser-ablated and machined titanium implants after 5 months of healing using scanning electron microscopy and histomorphometry.

## Materials and methods

### Implants

**Figure 1:**
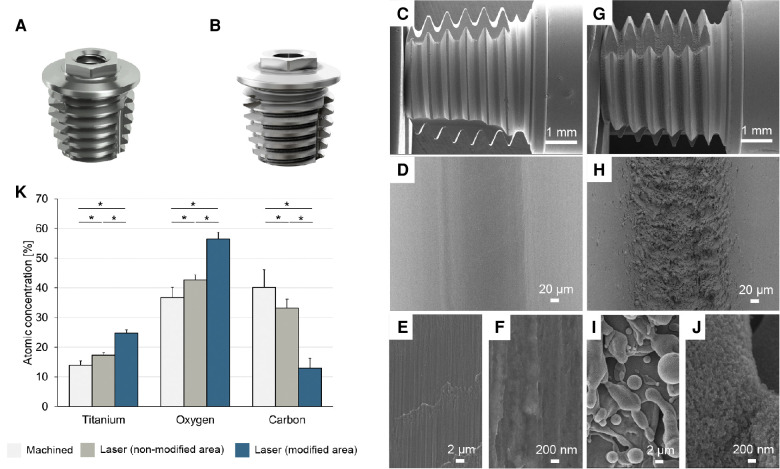
3d representation of the machined (**A**) and laser-modified (**B**) implants. Machined (**C–F**) and laser-modified implants (**G–J**) observed using secondary electron scanning electron microscopy. The surface of the machined implants exhibits ridges and grooves resulting from the machining process (**C–F**) whereas a distinct micro- and nanotexture is superimposed in the thread valleys of the laser-modified implant (**G–J**). Chemical composition of the surfaces of the machined implant and the nontreated and treated areas of the laser-modified implant as determined by Auger Electron Spectroscopy (AES) (**K**).

Commercially available implants from Oticon Medical (Askim, Sweden), either Wide Ponto implants (machined, control) (Figure 1A) with a machined surface or Ponto BHX implants (laser, test) (Figure 1B) with a laser-ablated surface, were used in the current study, both with a diameter of 4.5 mm, length 4 mm and manufactured from commercially pure (c.*p*.) titanium grade 4.

Four implants of each type were chemically characterised using Auger Electron Spectroscopy (AES) (PHI 700 Scanning Auger Microprobe, Physical Electronics Inc., Chanhassen, Minnesota) operating at 3 keV. For each of the machined implants, measurements at four sites in two adjacent thread roots were made. Each of the laser-modified implants was analysed at two sites in a laser-treated area and two sites in a nontreated area. The surface morphology was thereafter characterised by scanning electron microscopy (Leo Ultra 55) operating at 5 kV.

### Animal surgery

This study was conducted in accordance with the OECD Good Laboratory Practice regulations, ENV/MC/CHEM (98) 17, with the European Good Laboratory Practice regulations, 2004/10/EC Directive, and with the United States Food and Drug Administration Good Laboratory Practice regulations, 21 CFR 58 with the exception of the housing during the follow-up period that was conducted using a non-GLP but audited and approved by Bergerie de la Combe aux Loups (ISO 9001 certified provider) and the bone-implant analyses that was performed by the Department of Biomaterials, University of Gothenburg, Sweden. The study protocol was reviewed and approved by the NAMSA Ethical Committee on September 19, 2016 (Study No. 213096). The article was written in accordance with the ARRIVE (Animal Research: Reporting In Vivo Experiments) 2.0 guidelines (Supplementary Material 1) (8).

A total of eight female sheep [*Ovis aries*, Strain/breed: Blanche du Massif Central (BMC)] each received one machined implant and one laser-modified implant bilaterally in the frontal cranial bone (*n* = 8). The animals were acclimatised for at least five days prior implantation. At the time of the implantation the sheep were 3.8 ± 0.1 years old (mean ± SD) and had body weights of 71 ± 4 kg (mean ± SD). Only healthy, previously unused animals weighing above 60 kg were allowed to be included in the study. The day before implantation, the sheep were weighed and administered antibiotics (amoxicillin (Duphamox LA®, Zoetis, intramuscular (IM) and enroftoxacin, Baytril® 10%, Bayer Pharma, IM or subcutaneous (SC)). The sheep were fasted prior to surgery. Premedication was performed by intravenous (IV) injection of a mixture of diazepam (Valium®, Roche) and butorphanol (Torphasol®, Axience). Anesthesia was induced by IV injection of propofol (Propovet®, Axience) and maintained by inhalation of an O_2_-isoflurane mixture (Isoflo®, Axience, 2 to 5% through a tracheal tube). Each sheep preoperatively received a nonsteroidal anti-inflammatory drug (flunixine, Meflosyl® Injectable, Zoetis, IM). As a prophylactic measure, a perioperative antibiotic (enrofloxacin (Baytril® 10%, Bayer Pharma), SC or IM) was given.

An incision was made down to the bone at the parietal bone, and a flap was folded anteriorly using a self-retaining retractor to expose the implantation site. The positions for the test and control implants were marked with a sterile pen approximately 1 cm lateral to the sagittal suture and 1 cm anterior to the lambdoid suture (frontal-parietal suture). The machined and laser implants were randomised to either side of the sagittal suture. The osteotomies were prepared with stepwise drilling using the MIPS technique according to the manufacturer's instructions (9). Proper seating was confirmed visually, and insertion torque noted. Manual tightening was performed if needed. Cover screws were then attached to the implants before closing the incision in layers using absorbable sutures (Vicryl® 2.0 or 3.0, ETHICON) for the subcutaneous and intradermic tissues and absorbable sutures (Vicryl® 3.0, ETHICON for three sheep) or nonabsorbable sutures (Prolene® 2.0, ETHICON for five sheep) for the cutaneous tissue. The wounds were disinfected using oxytetracycline (Oxytetrin® spray, MSD). The sheep were thereafter allowed to recover from anesthesia in the operating room and returned to their individual pens, the supply of food and water was reinstated, and the sheep were kept under close observation. Buprenorphine (Buprecare®, AXIENCE) was given after the surgery and then twice daily for 2 days post-surgery (IM). An antiinflammatoryv drug (flunixine, Meflosyl® Injectable, Zoetis, IM) was given daily for 7 days post-surgery and antibiotics were given for 2 weeks post-surgery (amoxicillin, Duphamox LA®, Zoetis, IM, every 2 days and enrofloxacin, Baytril® 10%, BAYER PHARMA, SC or IM, daily). The sutures were removed after complete healing. The wounds were disinfected every 2 days with oxytetracycline (Oxytetrin® spray, MSD) until suture removal or 48 h after suture removal. During the study, the sheep were grouped and housed in pens, identified by an individual tag in the ear and a card indicating the study number, the sheep number, gender and the surgery date (NAMSA, Chasse-sur-Rhône, France). During the follow-up period, sheep were group housed in a farm setting (Bergerie de la Combe aux Loups, France), identified by an individual tag in the ear and a card indicating the study number, sheep number, gender and the procedure date. The animals were observed daily to detect mortality, morbidity or any clinical abnormality.

Five months (21 weeks) after the surgery, the sheep were euthanized by intravenous overdose of pentobarbital (Dolethal®, Vetoquinol). After exposing the area *via* an incision and removing the cover screws, the implants were retrieved together with the surrounding bone tissue *en bloc* using a 10 mm trephine drill and immersion fixed in 10% neutral buffered formalin. Due to the difference in design of the test and control implant the investigators performing the surgeries and analysis could not be blinded to the implant type.

### Sample preparation

Bone-implant samples were fixed in 10% neutral buffered formalin at pH 7.0 ± 0.1 for 3 days, dehydrated in a graded ethanol series, and resin embedded (LR White, London Resin Co. Ltd, UK). Resin embedded bone-implant blocks were bisected by sawing to prepare one central ground section ∼40 µm in thickness (Exakt Apparatebau GmbH & Co, Norderstedt, Germany) and stained with toluidine blue. The remaining half-blocks were wet polished using 400–4000 grit SiC paper for subsequent analyses.

### Histology and histomorphometry

Light optical microscopy (Nikon Eclipse E600) was used to assess the osseointegration and amount of the tissues surrounding the implant. Quantitative histomorphometry (Nikon NIS-Elements software) was performed to determine the amount of BIC and BA within the implant threads.

### Backscattered electron scanning electron microscopy

Polished, resin embedded bone-implant blocks were air-dried overnight prior to low-vacuum backscattered electron scanning electron microscopy (BSE-SEM) imaging in a Quanta 200 environmental SEM (FEI Company, The Netherlands) operated at 20 kV and 0.5 Torr water vapour pressure.

### Raman spectroscopy

Micro-Raman spectroscopy was performed using a confocal Raman microscope (Renishaw inVia™ Qontor®) equipped with a 633 nm laser and LiveTrack™ focus-tracking technology. The laser was focused down onto the sample surface using a × 100 (0.9 NA) objective. The Raman scattered light was collected using a Peltier-cooled CCD deep depletion NIR enhanced detector behind 1,800 g mm^−1^. Two regions of interest were defined: (i) within thread (mineralized bone within the first endosteal thread, i.e., below the level of the original cortical bone, to ensure that only *de novo*-formed bone was analysed) and (ii) native bone (original cortical bone ≥1 mm from the implant surface). From each region of interest, 6 to 8 spectra were collected (8 s integration time and 10 accumulations per spectrum). Background subtraction and cosmic ray removal were performed in Renishaw WiRE 5.2 software (10).

### Statistical analysis

Data was analysed by using SPSS Statistics for Windows (v27.0, IBM Corp) and Excel (v2021, Microsoft). Independent samples *t* tests were used to determine significant differences in surface purity and oxide thickness between the two implant types. The nonparametric Wilcoxon signed rank test was used for histomorphometric analysis and Raman spectroscopy, and *p* values <0.05 were considered statistically significant. Mean values ± standard deviations are presented.

## Results

Scanning electron microscopy of the machined implant revealed a relatively smooth surface with characteristic ridges and valleys created by the machining process (Figures 1C–F), whereas a distinct micro- and nanotexture was superimposed in the thread valleys of the laser-modified implant (Figures 1G–J). The surface of both implant types consisted predominantly of titanium, oxygen and carbon on both implant surfaces with minor impurities of calcium, phosphorous and sulfur, as revealed by the chemical analysis (Figure 1K). For the laser-modified implant, there was a significantly higher content of titanium and oxygen than on the machined implant. Furthermore, the laser-treated implant contained significantly less carbon than the machined implant. Similarly, the laser-treated roots of the thread contained significantly more titanium and oxygen and less carbon than the nontreated area of the implant threads.

**Figure 2:**
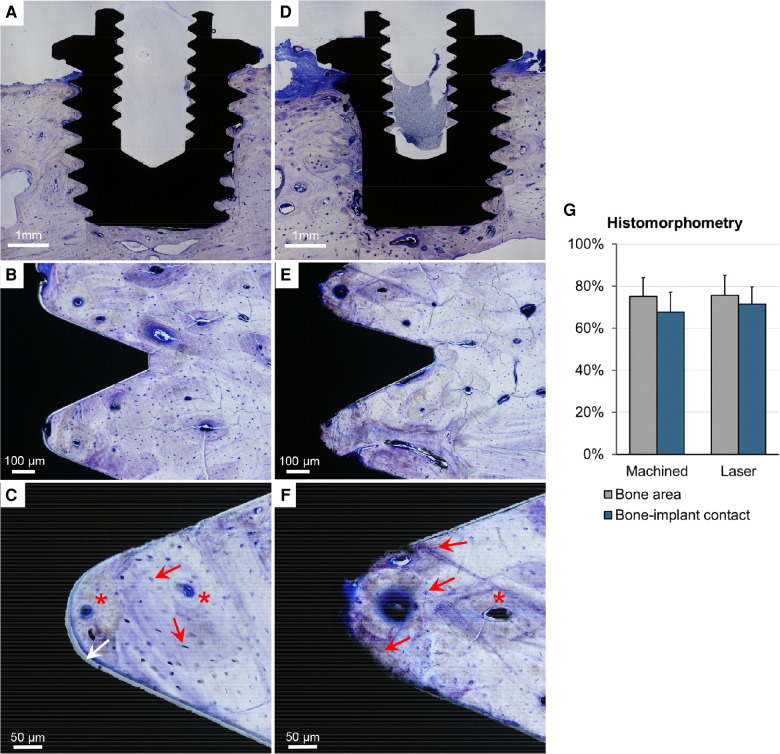
Integration between the bone and machined (**A–C**) and laser-modified (**D–F**) implants. Optical microscopy. Histological evaluation shows that both the machined (**A**) and laser-modified (**D**) implants are positioned vertically in the skull and surrounded by a considerable amount of bone tissue. No signs of inflammation were observed. Mature bone is detected close to the implant surface. Osteons with central blood vessels (red asterisk) and concentric lamellae with coaligned osteocytes (red arrow) are indicated (**C,F**). Separation of the bone tissue from the titanium surface (white arrow in **C**) is observed more frequently for the machined implants than for the laser-modified implants. (**G**) Bone area and bone–implant contact measured using histomorphometry.

No animals were excluded from the analysis. The animals healed uneventfully, and there were no adverse reactions either during the initial healing or at termination. The weight of the animals at explantation was 69 ± 7 (mean ± SD). In total *n* = 8 control implants (machined) and *n* = 8 test implants (laser) were retrieved and analysed. The general histological observation of the ground sections was that a large amount of bone had grown around and into the threads, with a mature, lamellar appearance (Figures 2A–F). Histomorphometry demonstrated substantial amounts of bone occupying the threads of the machined (75.1 ± 9.0%) and laser-modified (75.6 ± 9.5%) implants (Figure 2G). Direct bone-implant contact was also similar between the machined (67.7 ± 9.4%) and laser-modified (71.5 ± 8.2%) implants. There was no statistically significant difference between the two implant types in either bone area within the threads (*p* = 0.724) or bone-implant contact (*p* = 1.00).

BSE-SEM revealed high mineral content, and therefore maturation, of the peri-implant bone. The typical morphological features of remodelled lamellar bone, such as the presence of osteons and osteocyte lacunae aligned parallel to the implant surface, corroborated the histological observations.

**Figure 3:**
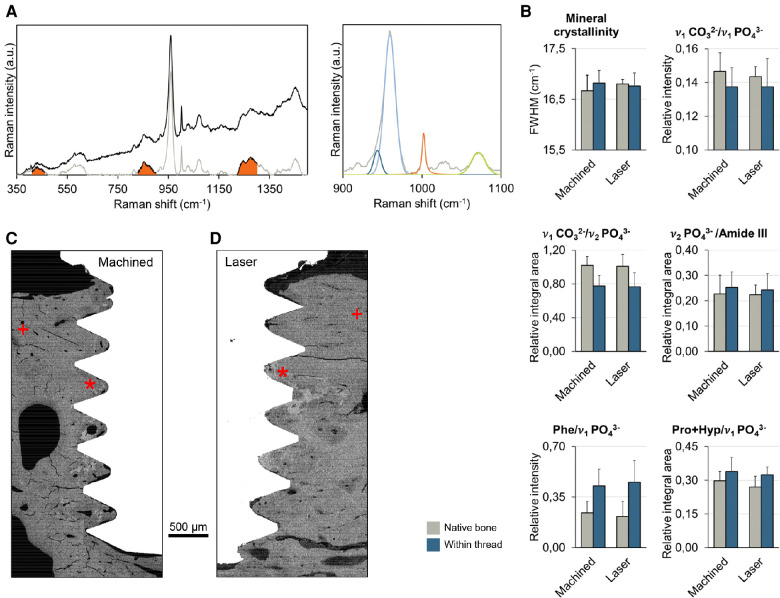
(**A**) Raman spectroscopy. *Left*: Before (black) and after (grey) background subtraction. Integral areas of *ν*_2_ PO_4_^3−^, Pro + Hyp, and Amide III are indicated. *Right*: Two Gaussian curves are fitted to the 940–980 cm^−1^ region (*ν*_1_ PO_4_^3−^). The full-width at half-maximum (FWHM) of the Gaussian curve centred at ∼960 cm^−1^ is taken as mineral crystallinity. A single Gaussian curve is fitted to the 1056–1086 cm^−1^ region (*ν*_1_ CO_3_^2−^). A single Lorentzian curve is fitted to the 998–1008 cm^−1^ region (Phe). (**B**) Bone extracellular matrix composition (mineral crystallinity, carbonate-to-phosphate ratios, and mineral-to-matrix ratios) assessed using Raman spectroscopy. (**C–D**) backscattered electron scanning electron microscopy (BSE-SEM) of the machined (**C**) and laser-modified (**D**) implants. * indicates *within thread* region of interest + indicates the *native bone* region of interest.

Raman spectroscopy revealed that the peri-implant bone adjacent to the laser-modified surface was identical in composition and quality to that opposing the machined surface (Figures 3A,B). At 5 months postinsertion, the peri-implant bone interfacing both implant types was compositionally similar to the native bone. The lower carbonate content and higher phenylalanine (Phe) indicated a relatively younger bone at the interface compared to the native bone (Figure 3B) (11).

## Discussion

Previous preclinical evaluations of implants with site specific, laser-ablated implant surfaces have shown good ability to osseointegrate in the long bones of rabbits in both the short- and long-term in the tibia and femur (12, 13). A lighter version of the same implant surface modification showed good osseointegration in short-term healing in the long bone (7); however, knowledge of the harder, denser bone of the skull is lacking. Furthermore, the use of commercially available implants has not been previously evaluated *in vivo*. The aim of the current study was therefore to extend previous preclinical knowledge using a more clinically relevant model (sheep cranial bone) using a commercial implant design with a long-term follow-up of 5 months of healing.

The sheep skull is described as a relevant site to perform functional evaluation of materials (14). The sheep skull presents similarities with the human skull regarding bone thickness and anatomy, has historically been used to evaluate the implant/tissue interface and bone healing performance, and offers enough space to allow the use of implants having a clinically relevant size and design (15).

Modification of implant surfaces aims to alter the physicochemical properties to improve bone healing and load transfer (5). This can be achieved by changing the surface topography on either the micro- or nanoscale or by modifying the surface chemistry through biomimetic coatings or functionalization. Surface modification by laser processing generally does not introduce contaminants onto the surface (16). The localised elevation of temperature and reaction with ambient oxygen, however, results in a thicker oxide layer (7), which influences the osteoconductive behaviour and facilitates tissue bonding (17). Even though the surface oxide thickness was not determined for the implants used in this study, the laser machining processing parameters used were the same as those in the study by Shah et al. (7). Interestingly, the surface chemistry of the laser-modified implant was altered by laser processing in the non-modified areas, likely due to the increased bulk temperature of the implant during laser processing of the thread valleys.

Enhanced bone formation (18) and biomechanical fixation (19) of bone anchored implants has been made possible by the intentional roughening of surfaces resulting in microtopography modification. A rougher surface, on the microscale, is currently available with minor differences on all implant systems in the dental field and for bone-anchored hearing aid implants (20). Here, qualitative histological analysis demonstrated that both machined (Wide Ponto) and laser-modified (Ponto BHX) implants become osseointegrated in the sheep skull after five months. BSE-SEM corroborated these histological findings. The histological and histomorphometric evaluations of selective, laser-modified implants in animal models have all demonstrated superior or similar outcomes compared to machined implants (6, 12, 13). The results from this study are in agreement with a previous long-term rabbit study in which a large amount of bone growth was observed for both implant types. No difference in the amount of bone was observed at an early time point using this type of surface, while the biomechanics were largely improved (7). Moreover, earlier studies have shown that the improved biomechanical properties withstand time (6).

The overall geometrical design and intended use of the Ponto BHX implants are identical to those of the Wide Ponto implants already successfully used clinically. In contrast to the current, clinically used wide diameter implants (4.5 mm), previous generations were machined with a diameter of 3.75 mm. A recent review of the Ponto implant system (Oticon Medical AB) demonstrated an overall implant survival rate of 97.7% (21). This review did, however, include various implant types (narrow diameter, wide machined and wide with laser modification) in the evaluation. In adult patients, the three-year implant survival was 95.0% for the narrow 3.75 mm implant and 97.4% for the wider machined version (Wide Ponto) (22). In comparison, the survival rate of the Ponto BHX implant was reported to be 97% during a follow-up period of 15 months, which was also observed in an adult population (23). In comparison, the results from a retrospective clinical study on dental implants with a similar laser-ablated surface modification applied to the valleys of the threaded implant revealed a cumulative survival rate of 99.3% for 310 implants placed in 83 patients after a five-year period (24). Compared to the published literature (dental implants with moderately rough surfaces), this is a high survival rate, which indicates a successful implant in adults.

Although the survival rate of adult patients with wide-diameter BAHS implants is high, in specific patient groups, i.e., children or patients with compromised bone quality, the incidence of implant loss is much higher. In these demanding cohorts, the implant loss rate was a significant issue when using older generation 3.75 mm implants with a survival rate of only 86% (25) but was subsequently improved using wider diameter, more stable implants (26, 27). Recently, a study evaluating the outcome using the laser-modified Ponto BHX implant in a paediatric cohort demonstrated a further increase in the survival rate after 1 year to 96.6% (28). The laser-ablated titanium implant for bone-anchored hearing implantation has an enlarged contact area for osseointegration compared to the standard machined implant. The results of animal studies measuring the removal torque of implants treated with laser ablation after different time points all demonstrated a significant increase of more than 150% compared to machined-only implants (6, 7, 12, 13). This clearly demonstrates that a site-specific laser-treated implant has a higher biomechanical capacity in torsion than a machined smooth textured implant, which could be one of the main reasons for the improved survival rate, particularly in demanding cases.

## Conclusion

At long-term healing, implant surfaces modified by selective laser ablation show osseointegration and tissue response (in terms of amount of bone-implant contact, bone area, and bone quality) that is comparable to machined surface implants in a sheep calvarial model. This indicates safe and efficient clinical potential.

## Data Availability

The raw data supporting the conclusions of this article will be made available by the authors, without undue reservation.
